# Genome-Wide Identification of G Protein-Coupled Receptors in Ciliated Eukaryotes

**DOI:** 10.3390/ijms24043869

**Published:** 2023-02-15

**Authors:** Shuai Luo, Peng Zhang, Wei Miao, Jie Xiong

**Affiliations:** Key Laboratory of Aquatic Biodiversity and Conservation, Institute of Hydrobiology, Chinese Academy of Sciences, Wuhan 430072, China

**Keywords:** G protein-coupled receptor, genome wide identification, ciliate, evolution

## Abstract

G protein-coupled receptors (GPCRs) are the largest family of transmembrane receptors and play important roles in many physiological processes. As a representative group of protozoa, ciliates represent the highest stage of eukaryotic cell differentiation and evolution in terms of their reproductive mode, two-state karyotype, and extremely diverse cytogenesis patterns. GPCRs have been poorly reported in ciliates. In this study, we identified 492 GPCRs in 24 ciliates. Using the existing classification system for animals, GPCRs in ciliates can be assigned to four families, including families A, B, E, and F. Most (377 members) belong to family A. The number of GPCRs is extremely different in different ciliates; the Heterotrichea ciliates usually have more GPCRs than other ciliates. Parasitic or symbiotic ciliates usually have only a few GPCRs. Gene/genome duplication events seem to play important roles in the expansion of the GPCR superfamily in ciliates. GPCRs in ciliates displayed seven typical domain organizations. GPCRs in an ortholog group are common and conserved in all ciliates. The gene expression analysis of the members in this conserved ortholog group in the model ciliate, *Tetrahymena thermophila*, suggested that these GPCRs play important roles in the life cycle of ciliates. In summary, this study provides the first comprehensive genome-wide identification of GPCRs in ciliates, improving our understanding of the evolution and function of GPCR in ciliates.

## 1. Introduction

GPCRs play important roles in signal transduction and responses to extracellular stimuli. They also function in the regulation of cellular metabolism, hormone secretion, behavior and mood regulation, immune activity, and sensory activities, thereby playing important roles in the growth, development, and reproduction of organisms [1]. Scientists have designed several systems to classify the GPCR superfamily using different features, such as the clans, ligands to which they bind, and physiological and structural aspects. One popular system is using clans which classify the GPCRs into six families [2,3]: A (rhodopsin-like), B (secretin receptor family), C (metabotropic glutamate/pheromone), D (fungal mating pheromone receptors), E (cyclic AMP receptors), and F (frizzled/smoothed). This classification system was mainly designed to cover all vertebrate and invertebrate GPCRs. Although the sequence similarities among GPCRs have largely diverged, previous studies suggested that this superfamily may have originated and diversified in early eukaryotic evolution [4,5]. Due to their important roles, GPCRs have been widely identified and studied in many species, especially in model organisms. In humans, nearly 900 GPCRs have been identified. While 813 of these can be classified into four families, others cannot be assigned to A–F families at all. In *Drosophila*, more than 100 encoded proteins were originally considered to be GPCRs and have been classified into four families. With the rapid development of high-throughput sequencing technology, the genomes of more and more organisms have been sequenced successfully. The whole genome identification of GPCR has also been performed in multiple organisms, including *Homo sapiens* [6], *Gallus gallus* [7], *Rattus rattus* [8], *Mus musculus* [9], *Anopheles gambiae* [10], *Caenorhabditis elegans* [11], and *Drosophila melanogaster* [12].

Although GPCRs have been widely studied in multicellular organisms, little is known regarding their presence in unicellular organisms. Yeasts seem to encode a surprisingly small number of GPCRs. In *Saccharomyces cerevisiae* and *Saccharomyces pombe*, only three and nine GPCRs were found, respectively. An interesting question asks what might be the number and functions of GPCRs in unicellular organisms. The structural difference of GPCRs usually means a difference in function. In addition, the presence or absence of a certain type of GPCR in an organism may lead to functional differences between different organisms. A good example is the opsin receptors, which are designed to detect light and can be found in any animal [13,14]. However, the structure and number of opsin receptors varied considerably in Crustacea and led to differences in spectral sensitivity in Crustacean eyes, which are linked to ecological habitat and vitality [15]. Considering that GPCRs may have originated from the early evolution of eukaryotes, there may be some structural and functional differences in GPCRs between unicellular and multicellular organisms. In addition, the unique life cycle of unicellular organisms also suggests novel GPCRs in these organisms [16].

Ciliates are an important group of unicellular eukaryotes, common almost anywhere where water is present. In the microbial food webs of water bodies, ciliates have important functions because of their ecological responsiveness to various environmental stimuli [17,18,19]. In addition, some facultative and obligate parasitic ciliates cause widespread concern because they often lead to host diseases [20]. In general, ciliate cells as a whole need to respond and adapt to changes in the environment. Their GPCRs function in response to external stimuli and therefore play an important role in their life cycles. In ciliates, previous studies in *Tetrahymena thermophila* showed chemosensory responses to many different stimuli. As free-living organisms, *Tetrahymena* cells are able to change the speed and direction of their swimming and respond to different chemorepellents and chemoattractants [21,22,23,24]. The ability to respond to different chemicals allows these cells to swim away from hazardous areas and towards preferred locations in the water environment. In *Paramecium*, the chemoattractants also change their swimming speed. Intracellular electrophysiological measurements in *Tetrahymena and Paramecium* showed that they are generally similar. Therefore, these ciliates may incorporate sensory function and membrane potential changes to generate responses. Many sensory reception processes in eukaryotes usually involve the ligand activation of a G protein-coupled receptor [25,26]. Several studies have demonstrated that canonical GPCRs exist in ciliates, but the composition and function of GPCRs in ciliates are not yet clear [27,28,29]. In addition, ciliates exhibit diverse cell sizes, living environments, and life styles. Their GPCRs may vary significantly among different species.

In this study, we collected the genome data of ciliates in public databases, including ciliates from the Hymenostomatida [30], Euplotida [31], Sporadotrichida [32], Philasterida [33], Peniculida [34], and Sessilida and Heterotrichida [35]. We performed whole-genome identifications of GPCRs in these ciliates. The comprehensive identification of the GPCRs in ciliates will help us to understand the diversification and functions of the GPCRs in unicellular organisms.

## 2. Results

### 2.1. Identification of GPCRs in Ciliates

A total of 526 GPCRs were identified in the genomes of 24 ciliates and three outgroups. Table 1 shows the classification of 526 GPCRs based on the domain annotations. In general, the rhodopsin family is the largest GPCR family in the ciliates, with a total of 377 members.

We also attempted to annotate the GPCRs based on the A-F classification system used in animals. Protein domain annotations and BLASTP search results were used to assign the GPCRs into A–F families. For BLAST searches, all the GPCRs were BLASTP searched against the GPCRdb using an e-value cut-off of 1 × 10^−5^, and the A–F families were assigned based on the top hit in GPCRdb. Of the 526 GPCRs, 499 (94.8%) could be classified in families, and the rest of the GPCRs were labeled Unknown (Supplementary Table S1). In those 27 species, we found the GPCRs could be assigned to the A, B, E, and F families.

**Figure 1:**
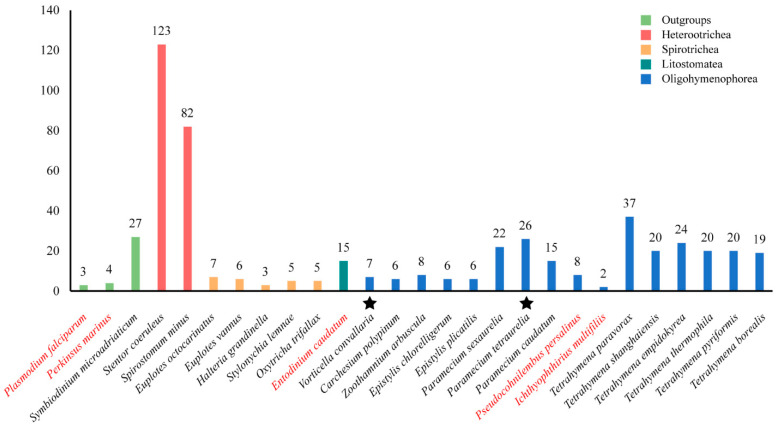
The number of GPCRs in 24 ciliates and three outgroups. Different colors represent different species. Red: parasitic and symbiotic species; black: free-living species. Stars indicate that the species have been shown to undergo genome duplication.

Figure 1 shows the number of GPCRs in different ciliate species. It seems that the number of GPCRs differs greatly in different species. *Stentor coeruleus* has the largest number of GPCRs with 123 members. The *Ichthyophthirius multifiliis* has the smallest number of GPCRs with only two members. In general, the class Heterotrichea ciliates, including *S. coeruleus* and *Spirostomum minus*, is shown to have the most GPCRs, followed by Oligohymenophorea (*Tetrahymena*, *Paramecium*, *Epistylis*, *Carchesium*, *Vorticella*, *Zoothamnium*), Spirotrichea (*Euplotes*, *Halteria*, *Oxytricha* and *Stylonychia*), and Litostomatea (*Entodinium*). Another interesting result was that the number of GPCRs identified in free-living ciliates was higher than that in parasitic or symbiotic ciliates, such as the fish obligate parasite *I. multifiliis* and the rumen anaerobic ciliate *Entodinium caudatum*.

### 2.2. Distribution GPCR Families in Ciliates

**Figure 2:**
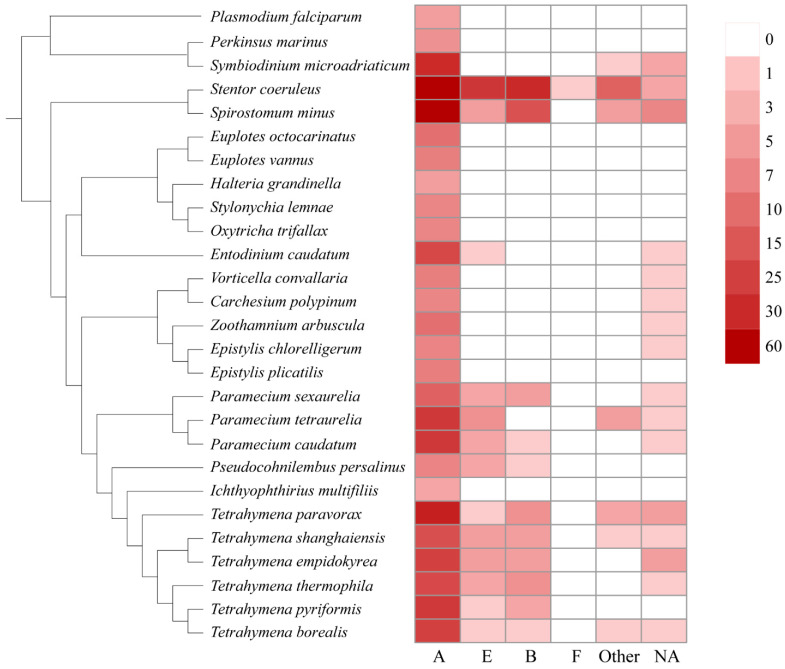
Distribution of different GPCR families in ciliates.

In this study, we also assigned the GPCRs to the existing classification system, e.g., the A–F families. As shown in Figure 2, the family A is the largest GPCR family in general, followed by families E, B, and F. No GPCRs belonging to families C and D were found in ciliates. Family A GPCRs seem to be most important in ciliates because they make up the largest GPCR family of all the ciliates (Figure 2). Family B and E GPCRs were mainly identified in *Paramecium*, *Tetrahymena*, *Stentor*, and *Spirostomum*.

Previous studies showed that family A rhodopsin-like GPCRs are the largest subfamily of G protein-coupled receptors and make up about half of all GPCRs, including hormones, neurotransmitters, and light receptors [36]. In general, rhodopsin-like GPCRs family members are complex and difficult to classify based on single characteristics, such as structure, function, and expression distribution, but most of these proteins transduce extracellular signals through coupled guanine nucleotide-binding (G) proteins [37]. These signals can be light, smells, ions, hormones, etc. In ciliates, family A GPCRs also had transmembrane-protein-GPR107-GPR108-like (IPR009637), phosphatidylinositol-4-phosphate-5-kinase (IPR023610), archaeal-bacterial-fungal-rhodopsins (IPR001425), intimal-thickness-related-receptor-IRP (IPR019336), and GPCR-rhodopsin-like-7TM domain (IPR017452). In previous studies, these domains were considered to play the role of retrograde transport in trans-Golgi networks [38,39], the phosphorylation of phosphatidylinositol-4-phosphate precursor in the phosphoinositide signaling pathway [40], light-dependent ion transport and sensory functions [41,42], and intimal thickening in mice and transduce extracellular signals.

Family B GPCRs have been widely identified in animals [43] but not in plants, fungi, or prokaryotes. In ciliates, family B GPCRs are mainly distributed in *Stentor*, *Paramecium*, and *Tetrahymena.* Family B GPCRs are mainly secretory, adherent, and responsible for both maintaining homeostasis and regulating behavior [44] such as glucose homeostasis, learning and memory, and stress-related autonomic, neuroendocrine, and behavioral function [45,46].

Family E GPCRs mainly consist of cyclic AMP receptor (CAR) proteins [47,48]. The cAMP receptors belong to the cell-surface receptor family. These receptors are usually involved in chemotaxis, aggregation, and morphogenetic movement [49]. In ciliates, family E GPCRs are mainly distributed in class Heterotrichida, Peniculida, and Hymenostomatida. An interesting phenomenon is that the distribution of family E GPCRs in ciliates coincides with the speed of swimming or the strength of the locomotion ability of ciliates [50]. *Paramecium* and *Stentor* have strong swimming ability, and a large number of family E GPCRs have been identified in their genomes. In contrast, ciliates in Sessilida and Spirotrichea had relatively weak swimming ability, and few or no family E GPCRs were identified in them.

Neither family C nor family D GPCRs were identified in all ciliates. Only one family, F GPCR, was identified in all the ciliates. Family D GPCRs were only identified and involved in fungi mating [51]. Family C and F are metabotropic glutamate receptors and frizzled and smoothened receptors, respectively. Family C GPCRs were reported to function in the central nervous system and in regulating Ca^2+^ homeostasis, while family F GPCRs are mainly involved in ontogeny and tissue homeostasis [52,53,54]. Our results suggest that these GPCR families are lacking in ciliates.

### 2.3. Protein Domain Architectures of GPCRs in Ciliates

**Figure 3:**
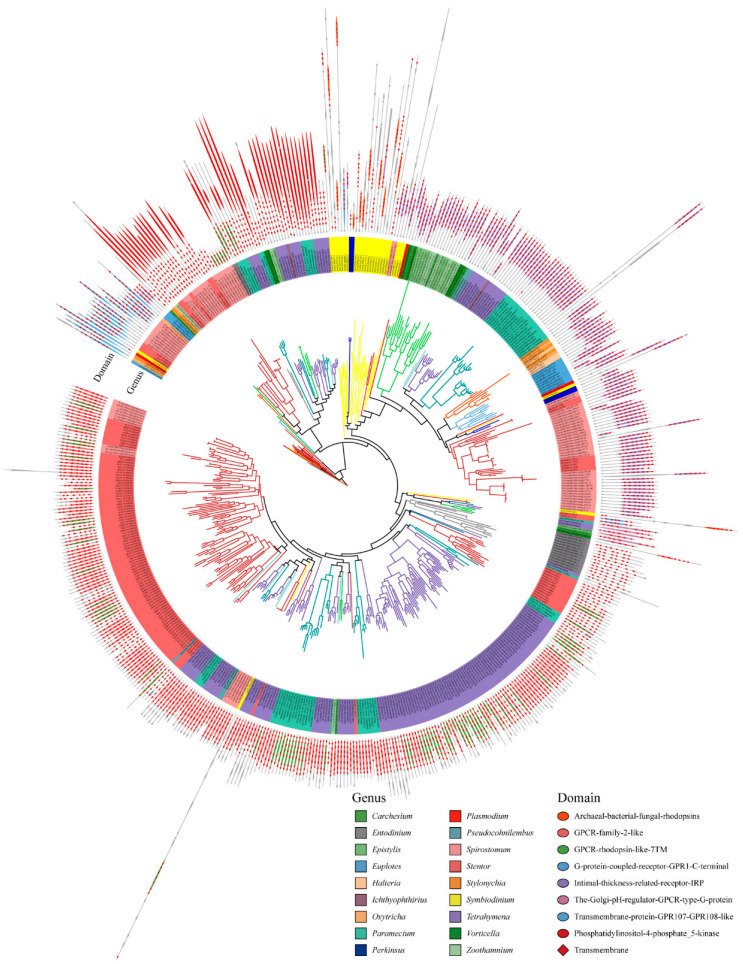
Phylogenetic tree and domain architectures of the GPCRs in ciliates.

We used the maximum likelihood method to construct a phylogenetic tree of GPCRs, and the domain organizations of each protein was assigned (Figure 3). The GPCRs in ciliates showed seven typical domain organizations: Types 1 to 7. These include Type 1, which represents the 5-8TMs + archaeal-bacterial-fungal rhodopsin (IPR001425); Type 2: 4-7TMs + GPCR family 2-like (IPR017981); Type 3: 3-8TMs + GPCR rhodopsin-like 7TM (IPR017452); Type 4: 6-7TMs + G protein-coupled receptor GPR1 C-terminal (IPR022596); Type 5: 3-8TMs + intimal thickness related receptor (IPR019336); Type 6: 3-8TMs + the Golgi pH regulator-GPCR-type G protein (IPR015672); and Type 7: 5-8TMs + transmembrane protein GPR107-GPR108-like (IPR009637). In addition, some GPCR also have accessory domains, e.g., phosphatidylinositol-4-phosphate 5-kinase (IPR023610).

**Figure 4:**
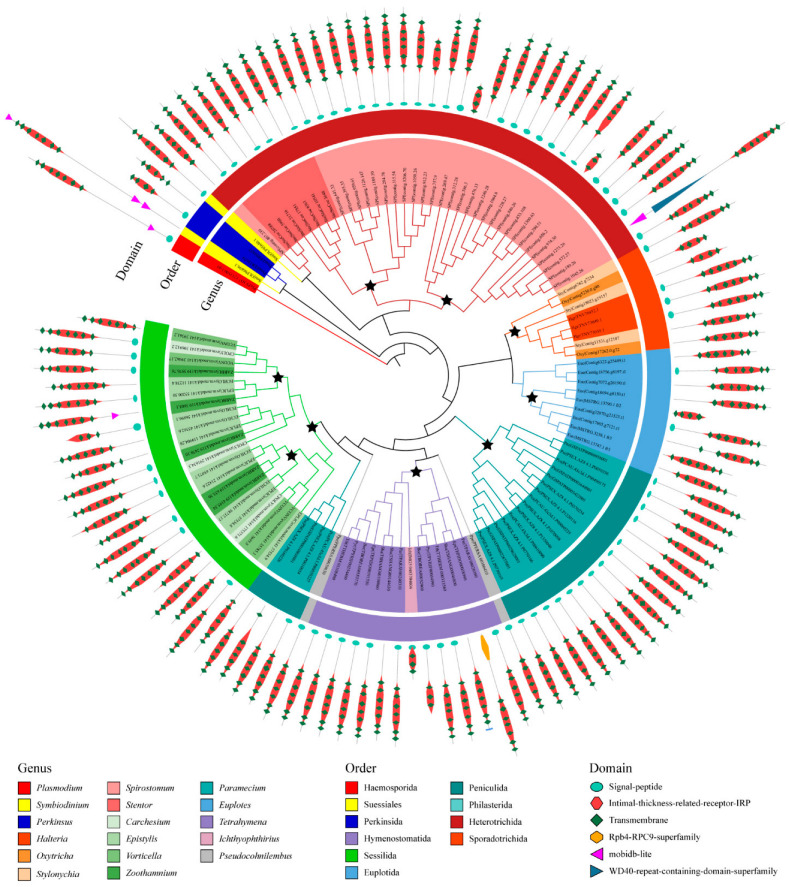
Phylogenetic tree of GPCRs in the most conserved ortholog group. Stars indicate the duplicate on events of GPCR genes.

We also divided the 526 GPCRs into ortholog groups using the OrthoFinder, and 84 ortholog groups were identified (Table S1, Figure S1). In general, the ortholog groups are lineage-specific (Figure S1). Among these 84 ortholog groups, only one was seen to exist in all ciliate species, suggesting that this ortholog group included the most common and conserved GPCRs in ciliates and may be involved in certain fundamental functions. In this conserved ortholog group, 115 GPCRs belonged to family A GPCRs and contained a protein domain PF10192. GPCRs in this ortholog group were similar in length and domain architecture. These GPCRs have an average length of 400 amino acids and usually have six to seven transmembrane helices. The phylogenetic analysis of GPCRs in this ortholog group suggested that many gene duplication events occurred (Figure 4). Most of the duplication events of GPCR genes commonly took place in the species or genus. For example, the ortholog group of GPCR is divided into several distinct branches in *Spirostomum minus*, and the GPCR is divided into two distinct categories in the genus *Tetrahymena*. Therefore, gene/genome duplication events seem to play important roles regarding the expansion of the GPCR superfamily in ciliates.

**Figure 5:**
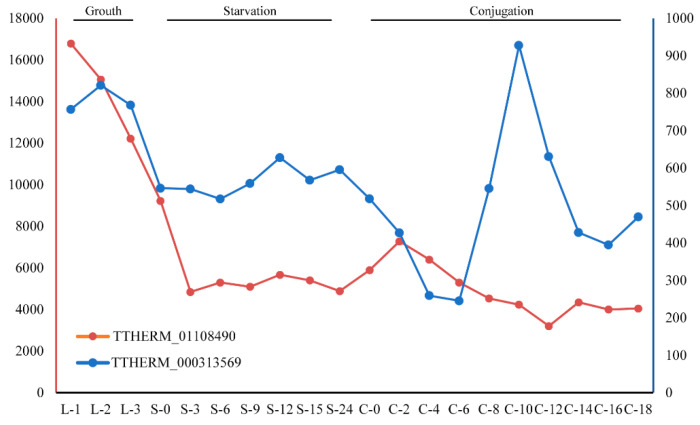
Expression levels of gene TTHERM_01108490 and TTHERM_000313569 at different stages of the life cycle in *Tetrahymena thermophila*.

The InterProScan annotation of these GPCRs demonstrated that most have the signal peptide and are annotated as intimal thickness related receptor (ITR, IPR019336). In mice, ITRs are usually expressed in vascular smooth muscle cells. ITR knockout mice were shown to be resistant to experimental intimal thickening, suggesting that ITRs play key roles in signal receiving in vascular smooth muscle cells [55]. ITRs usually have a domain with seven transmembrane alpha helices and belong to the rhodopsin-like GPCR superfamily [56]. This type of receptor could respond to many extracellular signals, such as hormones and lipid messengers. In the model ciliate, *Tetrahymena thermophila*, two genes belong to this ortholog group: TTHERM_01108490 and TTHERM_000313569. Gene expression analysis for these two genes showed that TTHERM_01108490 genes have high expression levels in all three stages (growth, starvation and conjugation) in the *Tetrahymena* life cycle, but especially in growth (Figure 5). The gene TTHERM_000313569, although displaying a relatively lower expression level than TTHERM_01108490, was also expressed in all three stages in the *Tetrahymena* life cycle. This result suggested that GPCRs in this ortholog group may play important roles in the life cycle of ciliates.

## 3. Discussions

### 3.1. High Variation of the Number of GPCR in Ciliates

Previous studies have shown significant differences in the number of GPCRs in multicellular animals. However, in ciliates, the number of GPCRs identified in different species can still differ by one to two orders of magnitude, which is very interesting. We speculate that three main factors affect the number of GPCR in ciliates. First, the large number of GPCRs in Heterotrichea ciliates may be related to the size and complexity of their cells. *S. coeruleus* is a large ciliate that is 0.5 to 2 mm in length. *S. minus* is 0.6 to 0.7 mm in length. These giant cells have complex cellular structures [57,58,59,60,61], and the large number of GPCRs may support their responses to the environment. Second, the number of GPCRs may be related to the lifestyle of ciliates. In this study, the result is similar to the finding in the Apicomplexa parasite, of which only several GPCRs have been characterized [62], and may reflect the relatively stable environments of these species encountered in its life cycle. Third, the number of GPCRs in ciliates may be related to gene/genome repetition events. Early studies demonstrated that the sequence similarity is minimal among distant GPCR proteins; thus, the origins of GPCRs between families were thought to be uncorrelated [63]. The identification and classification of GPCRs indicated a common evolutionary origin for all groups, and they may have arisen from a single ancestor through gene duplications. A study of *Caenorhabditis elegans*, *Drosophila melanogaster*, and *Anopheles gambiae* also suggested that GPCRs have ancient origins. Different GPCR members may have evolved through gene/genome duplications and in tandem with increasing organismal complexity [64]. Therefore, we investigated the relationship between the predicted proteome size and the number of GPCRs in ciliates. The effect of gene/genome repetition events and the number of GPCRs was positively correlated (*R* = 0.520, *p* = 0.005) (Figure S2, Table S2). Among the ciliates investigated in this study, genome duplication events have been reported in *Vorticella convallaria* and the *Paramecium tetraurelia*; the numbers of GPCRs identified in these two species are higher than those in other ciliates in the same order. These results suggested that gene/genome duplications may play important roles in the expansion of the GPCR superfamily.

### 3.2. Clues for the Function of GPCRs in Ciliates

The GPCRs are the largest family of transmembrane receptors, and most belong to the Family A GPCRs [3]. The rhodopsin receptor family, which is composed of Family A GPCRs, can be further divided into several subclasses, such as amines, peptides, proteins, lipids, sensory subclasses, and others. These play a crucial role in signal transduction [65]. Family A GPCRs are highly differentiated among species. However, in ciliates, an ortholog group of GPCRs was found that showed significant homology between species, making it the most common and conserved group among GPCRs. This ortholog group of GPCRs may be involved in regulating the cell life cycle, sexual partner recognition, and finding food [66]. In the model ciliate, *Tetrahymena thermophila*, there are two genes belonging to this ortholog group that have high expression levels in the growth or conjugation in the life cycle of *Tetrahymena*. Therefore, they may be involved in recognizing certain pheromones released during cellular mating. Despite the lack of clear functional annotations for many of the identified putative GPCRs, some of these genes have been classified as responsive to pheromones based on their gene ontology (GO) annotations. In particular, a gene in *Paramecium* and *Stylonychia* was annotated as the G protein-coupled receptor 180 (GPR180) [67,68]. Although its function has not yet been verified in ciliates, it has been shown to play a crucial role in signal transduction during gametogenesis in *Plasmodium*. In another study, the knockout of GPR180 had no observable impact on the blood development stage, but it did impair the formation of gametes [69]. This finding further confirms our conjecture that this class of GPCR may play an important role in the growth cycle or the mating of ciliates. However, more experimental data are needed to understand the detailed molecular functions of such GPCRs in ciliates.

## 4. Materials and Methods

### 4.1. Ciliate Genome Data Collection

To identify the GPCRs of ciliates, we collected the genome data of 24 ciliates, and the dataset was derived from four sources. Of the collected ciliates, the data for *Ichthyophthirius multifiliis*, *Tetrahymena borealis*, *Tetrahymena empidokyrea*, *Tetrahymena paravorax*, *Tetrahymena pyriformis*, *Tetrahymena shanghaiensis*, *Tetrahymena thermophila*, *Euplotes octocarinatus*, *Euplotes vannus*, *Stentor coeruleus*, *Oxytricha trifallax*, and *Stylonychia lemnae* were obtained from Ciliates.org (https://ciliates.org/landing/ (accessed on 12 October 2022)). The data for *Entodinium caudatum*, *Carchesium polypinum*, *Halteria grandinella*, *Pseudocohnilembus persalinus*, and *Spirostomum minus* were retrieved from NCBI (www.ncbi.nlm.nih.gov (accessed on 12 October 2022)). The data for *Paramecium caudatum*, *Paramecium sexaurelia*, and *Parameciium tetraurelia* were obtained from ParameciumDB (https://paramecium.i2bc.paris-saclay.fr/ (accessed on 12 October 2022)). Further ciliate data, including data for *Epistylis chlorelligerum*, *Epistylis plicatilis*, *Vorticella convallara*, and *Zoothamnium arbuscula* can be accessed through the National Genomics Data Center (https://ngdc.cncb.ac.cn/ (accessed on 12 October 2022)). This dataset covers four classes, eight orders, twelve families, and fifteen genera. In addition, we also identified the GPCRs in three non-ciliates species (used as outgroups), including *Perkinsus marinus*, *Symbiodinium microadriaticum*, and *Plasmodium falciparum*. These data were also obtained from NCBI (www.ncbi.nlm.nih.gov (accessed on 17 October 2022)).

### 4.2. Identification of Putative GPCR Genes

**Figure 6:**
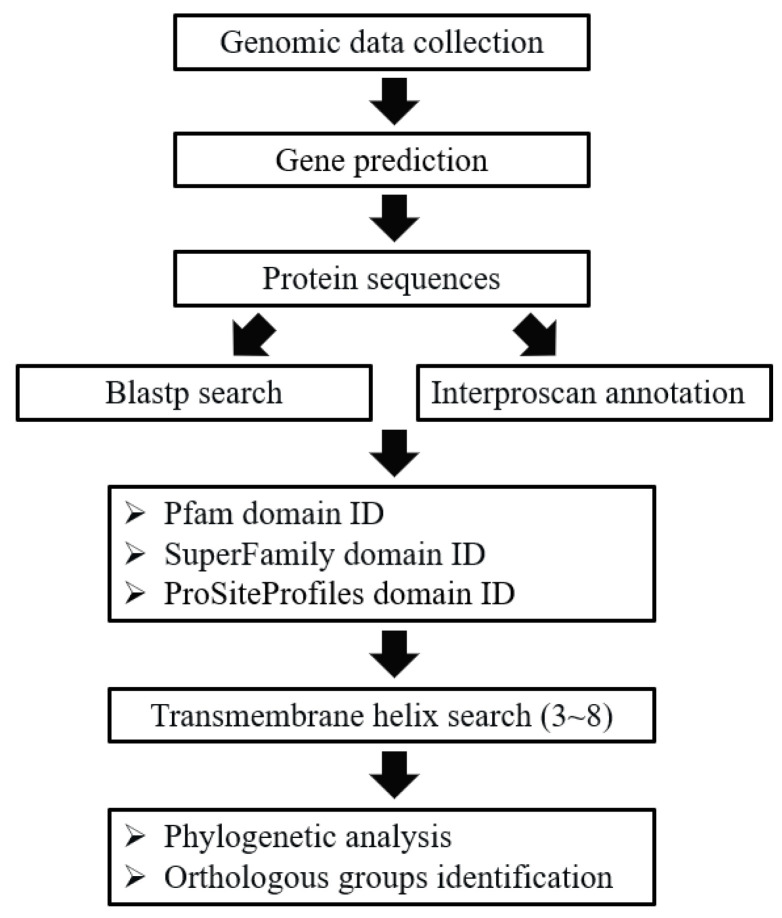
Pipeline used to identify GPCRs in ciliates.

In general, the GPCRs in ciliates were identified through a combination of BLAST and HMM searches. The procedure of GPCRs identification was described in Figure 6. To perform the BLAST searches, the GPCRs previously reported in *Bombyx mori* [70], *Caenorhabditis elegans* [71], *Drosophila melanogaster* [12], *Homo sapiens* [6], *Mus musculus* [9], *Arabidopsis thaliana* [72], and *Tetrahymena thermophila* [55] were collected and used as the seeds for BLASTP searches. To identify putative GPCRs sequences from protein sequences in collected genomes, BLASTP (version 2.6.0+) searches were first performed with a cut-off e-value of 1e-05 in order to search for all GPCRs candidates [73]. To identify putative GPCRs that lack significant sequence similarity to known GPCRs which may have been missed in the BLAST searches, more sensitive searches based on hidden Markov models (HMM) were performed using the InterProScan program [74,75], and protein domains were annotated. We used four independent domain annotations to screen the GPCRs in the collected genomes, including the Pfam domain, the Superfamily database, the PROSITE profiles database, and transmembrane helix information. For the Pfam domain, the proteins with 7TM_1/Rhodopsin (PF00001), 7TM_2/Adhesion (PF00002), 7TM_3/Glutamate (PF00003), Frizzled (PF01534), Ocular_alb (PF02101), Dicty_CAR (PF05462), Lung_7-TM_R (PF06814), GPCRsRhopsn4 (PF10192), Git3 (PF11710), GPR_Gpa2_C (PF11970), or ABA_GPCRs (PF12430) were selected as putative GPCRs; for the Superfamily database, Family A G protein-coupled receptor-like (SSF81321) was used; for the PROSITE profile database, G_PROTEIN_RECEP_F1_2 (PS50262) was used. All the candidates based on BLAST searches and protein domain annotation were combined. Finally, we used the transmembrane helix information (TMHMM [76]) to screen the GPCRs by requiring a protein to have three to eight transmembrane helices.

### 4.3. Identification of Ortholog Groups of GPCRs

To investigate the relationship of GPCRs in different ciliates, ortholog groups were identified using the predicted proteomes through the OrthoFinder (version 2.5.4) software (https://github.com/davidemms/OrthoFinder (accessed on 23 October 2022)). For phylogenetic analysis, multiple sequence alignment was performed using MAFFT (version 7.310) with E-INS_I parameter [77]. The most suitable substitution model was determined based on the Bayesian Information Criteria and Akaike Information Criteria [78]. The phylogenetic trees were constructed using RAxML (version 8.2.11) with the LG + G + F substitution model, and the reliability of the tree topology was evaluated with 1000 bootstraps [79]. The GPCR protein sequences, which were used for phylogenetic analysis, are provided in the Supplementary File S1.

## Figures and Tables

**Table 1:** Distribution of GPCRs in different protein domain categories.

Domain Id	Database	Database Annotation	GPCR Family	Numbers
PF00001	Pfam	7 transmembrane receptors (rhodopsin family)	A	18
PF00002	Pfam	7 transmembrane receptors (secretin family)	B	13
PF05462	Pfam	Slime mold cyclic AMP receptor	A, B, E, Other	120
PF06814	Pfam	Lung seven transmembrane receptor	A	22
PF10192	Pfam	Rhodopsin-like GPCRs transmembrane domain	A	133
PF11710	Pfam	G protein-coupled glucose receptor regulating Gpa2	Unknown	1
PF11970	Pfam	G protein-coupled glucose receptor regulating Gpa2 C-term	Unknown	9
PF12430	Pfam	Abscisic acid G protein-coupled receptor	Unknown	7
PS50262	ProSite	G protein-coupled receptors family 1 profile	Unknown	10
SSF81321	Superfamily	Family A G protein-coupled receptor-like	A, B, F, Other	193

## Data Availability

Data is contained within the article or Supplementary Material.

## Supplementary Materials

The following supporting information can be downloaded at: https://www.mdpi.com/article/10.3390/ijms24043869/s1.
