# Supplementary figures and images for: Genome-Wide Identification of G Protein-Coupled Receptors in Ciliated Eukaryotes
Source: Int J Mol Sci. 2023 Feb 15;24(4):3869. doi: 10.3390/ijms24043869 (PMC9960496; doi:10.3390/ijms24043869)

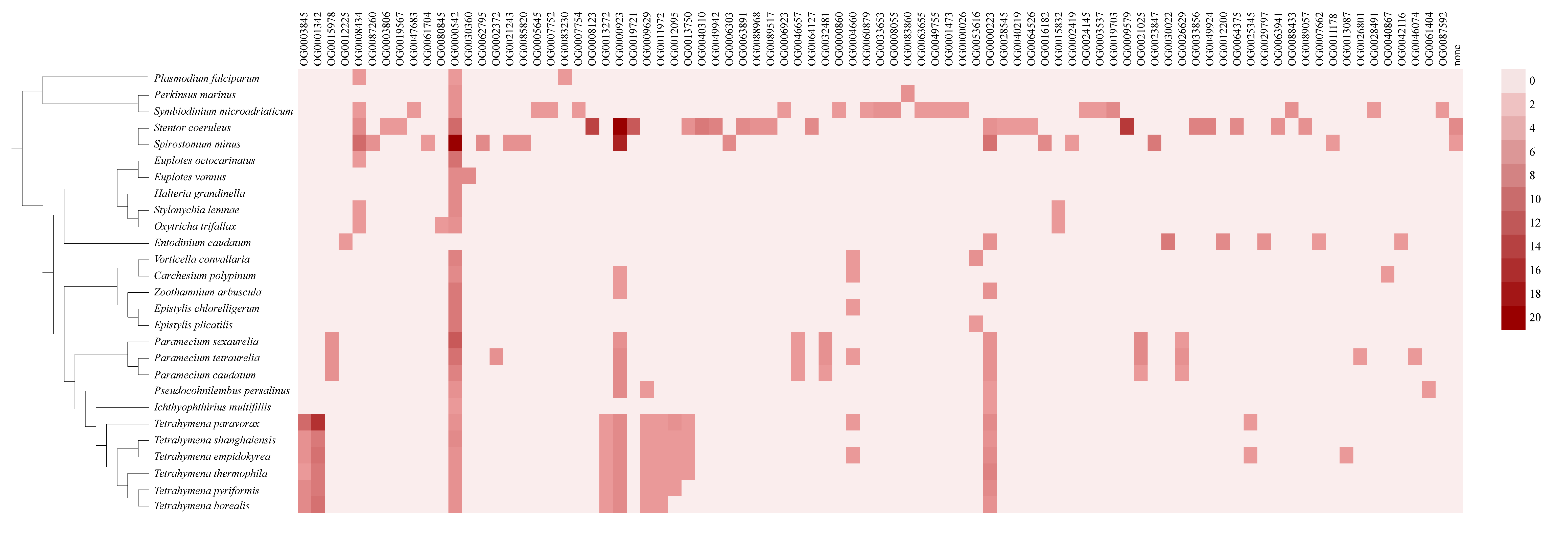

Supplement: Supplementary file 1 [file ijms-24-03869-s001.zip › Figure S1.tif]

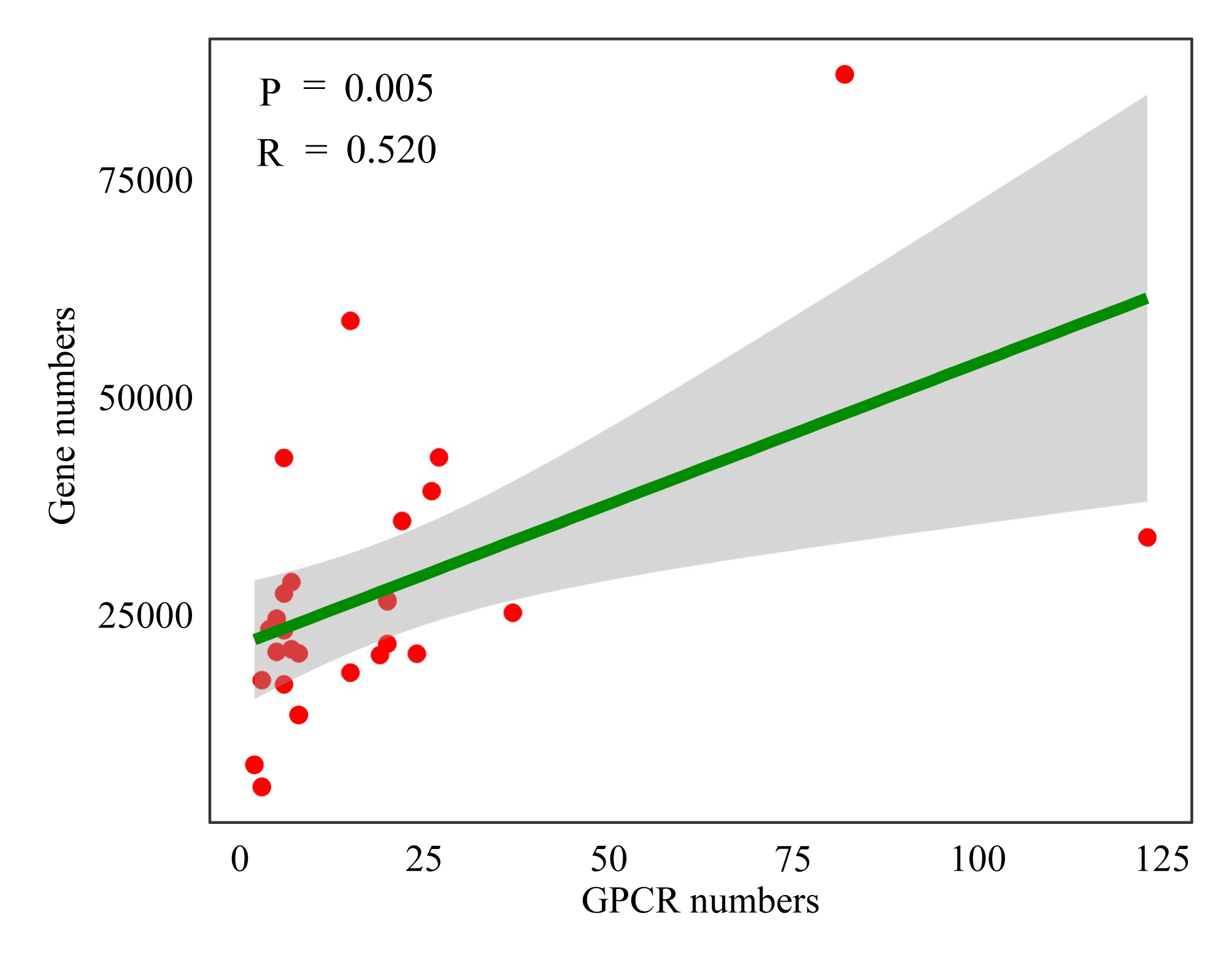

Supplement: Supplementary file 1 [file ijms-24-03869-s001.zip › Figure S2.tif]
